# SPAG5 promotes hepatocellular carcinoma progression by downregulating SCARA5 through modifying β-catenin degradation

**DOI:** 10.1186/s13046-018-0891-3

**Published:** 2018-09-18

**Authors:** Hongliang Liu, Junwen Hu, Ran Wei, Longfei Zhou, Hua Pan, Hongchao Zhu, Mingwen Huang, Jun Luo, Wei Xu

**Affiliations:** 1 Department of General Surgery, The Second Affiliated Hospital of Nanchang University, No. 1 Min De Road, Nanchang, 330006 Jiangxi Province China; 2 Department of Hepatobiliary Surgery, Tumor Hospital of Guanxi Medical University, Nanning, China; 3 Department of General Surgery, Third Affiliated Hospital of Nanchang University, Nanchang, China; 4 The First Clinical Medical College of Nanchang University, Nanchang, China; 5 Department of Rehabilitation Medicine, The Second Affiliated Hospital of Nanchang University, No. 1 Min De Road, Nanchang, 330006 Jiangxi Province China

**Keywords:** SPAG5, Hepatocellular carcinoma, Progression, SCARA5, β-Catenin, Degradation

## Abstract

**Background:**

The sperm-associated antigen 5 (SPAG5) plays a key role in controlling various cellular phenomena, including cell cycle progression and proliferation. However, the role of SPAG5 in hepatocellular carcinoma (HCC) remains unknown.

**Methods:**

This study investigated the function and clinical significance of SPAG5 protein expression in hepatocellular carcinoma. We analyzed SPAG5 expression in surgical specimens from 136 HCC patients. The correlation between the clinical characteristics and prognosis was also determined. Furthermore, the SPAG5 was overexpressed in HCC cell and silenced with shRNA in HCC cells. Moreover, cell proliferation and apoptosis were measured using Edu assay and flow cytometry and a molecular mechanism of SPAG5 promotes HCC progression was explored.

**Results:**

Herein, our study showed that upregulation of SPAG5 was detected frequently in primary HCC tissues, and was associated with significantly worse survival among the HCC patients. Multivariate analyses revealed that high SPAG5 expression was an independent predictive marker for the poor prognosis of HCC. SPAG5 silence effectively abolished the proliferation abilities of SPAG5 in vivo and in vitro, while induced apoptosis in HCC cells. Furthermore, our results indicate that SPAG5 promoted cell progression by decreasing SCARA5 expression, which has been reported to control the progression of HCC, and our data demonstrated that SCARA5 is crucial for SPAG5-mediated HCC cell progression in vitro and in vivo*.* Moreover, we found that the expression of SPAG5 and SCARA5 are inversely correlated in HCC tissues. In addition, we demonstrated that SPAG5 promoted progression in HCC via downregulating SCARA5 depended on the β-catenin/TCF4 signaling pathway. Interestingly, the underlying mechanism is which SPAG5 regulates SCARA5 expression by modulating β-catenin degradation.

**Conclusions:**

Taken together, our data provide a novel evidence for the biological and clinical significance of SPAG5 as a potential biomarker, and we demonstrate that SPAG5-β-catenin-SCARA5 might be a novel pathway involved in HCC progression.

**Electronic supplementary material:**

The online version of this article (10.1186/s13046-018-0891-3) contains supplementary material, which is available to authorized users.

## Background

Hepatocellular carcinoma (HCC), a major type of liver cancer, is the fifth most common cancer globally and ranks as the second leading cause of cancer death in males, among whom hepatocellular carcinoma (HCC) accounts for 70% to 85% of the total cancer burden [1, 2]. Despite current advances in the diagnosis of HCC, the majority of patients are not eligible for surgical treatment due to late diagnosis. Moreover, the five-year survival rate of patients undergoing surgical resection is disappointingly low [3]. Tumor proliferation and apoptosis plays an important role in the occurrence and development of HCC [4]. The researches have demonstrated that HCC proliferation and apoptosis are closely related to abnormal expression of oncogenes and tumor suppressor genes [5, 6]. Therefore, identification of novel diagnostic markers and therapeutic targets in HCC are urgently needed.

Sperm-associated antigen 5 (SPAG5) is associated with the mitotic spindle; during metaphase it is concentrated at the centromere [7, 8]. During mitosis, SPAG5, together with other proteins, forms a molecular switch at the centromere, jointly regulating the centromere-microtubule dynamics, and promoting mitotic processes and their fidelity. Thus, SPAG5 has an important role in the regulatory network of mitosis through interactions with a number of protein partners [9]. Recently, SPAG5 has been shown to be involved in tumorigenesis. It was reported that SPAG5 is amplified at the 17q11 region in some types of cancer. Overexpression of SPAG5 has been implicated in cancer growth and progression, and it is upregulated in breast cancer, lung cancer, bladder urothelial carcinoma and cervical cancer [10–13]. In addition, its overexpression is associated with poor patient survival. These studies have suggested that SPAG5 may play an important role in the tumorigenesis and progression of HCC. However, SPAG5’s biological role and clinical significance in HCC remains unclear.

Scavenger receptor class A member 5 (SCARA5) is a member of the scavenger receptor family and is associated with several human tumors [14–16]. The study found that SCARA5 expression is frequently downregulated in liver cancer, breast cancer, lung cancer and glioma. Recent studies have reported that SCARA5 is a tumor suppressor gene and plays an important role in the tumor progression processes [17, 18]. For example, the knockdown of SCARA5 can promote cell proliferation in renal cell carcinoma, whereas increased SCARA5 expression inhibits cancer proliferation [18]. Recently, our previous studies have demonstrated that SCARA5 knockdown can promote cell progression in HCC, whereas increased SCARA5 expression inhibits HCC progression [17]. However, the regulatory mechanisms of SCARA5 in HCC are not yet clear.

In this study, we first found that SPAG5 expression is upregulated in HCC tissues compared with that in non-tumor renal tissues, and its expression level exhibited a negative correlation with patient survival. Multivariate analyses revealed that high SPAG5 expression was an independent predictive marker for the poor prognosis of HCC. Furthermore, our results provided the first evidence that the downregulation of SPAG5 represses HCC proliferation by upregulating SCARA5 expression, while induced apoptosis in HCC cells. Moreover, our dates showed that SCARA5 is crucial for SPAG5-mediated HCC cell progression in vitro and in vivo. Mechanistically, we found that SPAG5 can regulate SCARA5 expression depend on β-catenin/TCF4 pathway in HCC cells. Mechanistically, SPAG5 regulates SCARA5 expression by modulating β-catenin degradation. Taken together, our data provide a novel evidence for the biological and clinical significance of SPAG5 as a potential biomarker, and we demonstrate that SPAG5-β-catenin-SCARA5 might be a novel pathway involved in HCC progression.

## Methods

### Clinical specimens

All primary HCC tissues and corresponding non-tumor tissues were obtained from 136 patients at the Department of General Surgery, The Second Affiliated Hospital of Nanchang University, between May 2010 and January 2017. All specimens were obtained during surgery, immediately frozen in liquid nitrogen, and stored at − 80 °C for further analysis. The identification of tumor tissues and adjacent normal tissues were confirmed by the pathologists. Written informed consent was obtained from each patient. This study was approved by the Ethics Committee of The Second Affiliated Hospital of Nanchang University.

### Cell culture

Human HCC cell lines (Huh7, HCCLM3, SMCC7721 and Hep3B) were purchased from the Shanghai Institute of Cell Biology, China. The immortalized liver cell line HL-7702 was purchased from Shanghai Fu Xiang Biotechnology Co., Ltd. The cells were cultured in DMEM (Gibco) supplemented with FBS (Hyclone) to a final concentration of 10% and were exposed to antibiotics at 37 °C with 5% CO_2_.

### Real-time quantitative polymerase chain reaction (qRT-PCR)

Total RNA was isolated from tissues and cultured cells using Trizol reagent (Invitrogen, USA) according to the manufacturer’s instructions. RNA was reverse-transcribed using the PrimeScript RT Reagent Kit (Invitrogen, USA). For quantitative polymerase chain reaction (PCR) analysis, aliquots of double-stranded cDNA were amplified using a SYBR Green PCR Kit (Applied Biosystems, Carlsbad, CA) and an ABI PRISM 7900 Sequence Detector (Applied Biosystems). qRT-PCR was performed on the cDNA using specific primers (Sangon, Shanghai, China) for SPAG5 and SCARA5. GAPDH was used as an internal control.

### Constructs and plasmids

The RNA duplexes for shRNA-mediated SPAG5, SCARA5 and β-Catenin silencing were synthesized by Genepharma Company (Shanghai, China). In addition, the plasmid of SPAG5, SCARA5 and β-Catenin were purchased from Genepharma Company. Transfections of the shRNA and plasmid in HCC cells were performed using Lipofectamine 2000 Transfection Reagent (Invitrogen, USA) following the manufacturer’s recommended protocol.

### Immunohistochemistry

Sections of HCC and tumor tissue of nude mice were treated with xylene and graded alcohol, and then subjected to antigen retrieval in 0.01 M citrate buffer. Hydrogen peroxide was used for blockage. The sections were incubated with goat serum for 30 min and then with anti-SPAG5 monoclonal antibodies (Sigma-Aldrich, 1:150 dilution) or anti-C-caspase3 (CST, 1:200) overnight at 4. A 2-step immunohistochemical method (catalog no.: PV-9000; ZSGB-BIO Co., Ltd., Beijing, China) was adopted for immunostaining. The staining intensity and percentage of positive cells were scored semi-quantitatively by 3 pathologists who were blind to the clinical parameters.

### 5-Ethynyl-20-deoxyuridine assay and cell counting kit-8 (CCK-8) assay

The cells were incubated with 5-ethynyl-20-deoxyuridine (EdU; Ribobio) for 5 h, and processed according to the manufacturer’s instruction. After three washes with PBS, the cells were treated with 300 μL of 1 × Apollo reaction cocktail for 30 min. Then, the DNA contents of the cells in each well were stained with 100 μL of Hoechst 33342(5 μg/mL) for 30 min and visualized under a fluorescence microscope.The Cell counting kit-8 (CCK-8) assay was performed as previously described [17].

### Cell-cycle analysis

1 × 10^6^ cells were trypsinized and washed with PBS twice and fixed with 1 ml pre-chilled 70% ethanol. The cells were washed with PBS twice. Then, the cells were incubated with propidium iodide (Sigma-Aldrich, St. Louis, MO, USA) and RNaseA for 30 min at room temperature and then for 30 min at 4 °C in the dark. A 300-mesh screen filter was used to filter the cell suspension and remove adhesive cells. Flow cytometry (BD Biosciences, San Jose, CA, USA) was used to analyze DNA content, and the software was used to estimate cell numbers in G_0_/G_1_, S, G_2_/M phases, and the proportions.

### Cell apoptosis analysis

The apoptosis rate of cells was tested by Apoptosis Detection Kits (BD, USA) following the manufacturer’s instructions. In brief, 5 × 10^5^ cells were harvested by centrifugation at 1000 g for 5 min and resuspended in 200 μL binding buffer, followed by a 15 min incubation with 5 μL Annexin V-FITC and 5 μL propidium iodide (PI) in the dark at 37 °C. Then, the flow cytometry analysis was employed for detecting apoptotic events.

### Tumorigenicity assay

1 × 10^6^ cells in 100 ml of PBS were injected subcutaneously into the flanks of nude mice (male BALB/c-nu/nu, 6–8 weeks, Shanghai SLAC Laboratory Animal Co., Ltd.). Tumor formation in nude mice was monitored, and the tumor volume was measured every 5 days. Tumors were harvested and individually weighed after the mice were anesthetized. The data are presented as tumor weight (mean ± SD).The animal work was approved by the Ethics Committee for Animal Experiments of the Second Affiliated Hospital of Nanchang University.

### Luciferase reporter gene assay

The cells were seeded at a density of 1 × 10^5^ cells per well in 6-well plates and incubated for 24 h before transfection with wild-type TOP-flash (2 mg) or mutant FOP-flash (2 mg) using LipfectamineTM LTX according to the manufacturer’s instructions. The pRL-SV40 expression vector (0.3 mg) was added to each transfection system to normalize the transfection efficiency. The reporter assays were performed according to the manufacturer’s instructions. The mean values of the normalized ratios were compared.

### Statistical analysis

All results are shown as mean ± SD and were analyzed using GraphPad Prism 6 (GraphPad Software, USA) from at least three independent experiments. The Kaplan-Meier method was used to calculate the survival curve, and log-rank test to determine statistical significance. The differences between groups were analyzed using Two-tail Student’s t test and ANOVA. Data were considered statistically significant when *p* < 0.05.

## Results

### SPAG5 is significantly upregulated in HCC tissues and cells

**Fig. 1:**
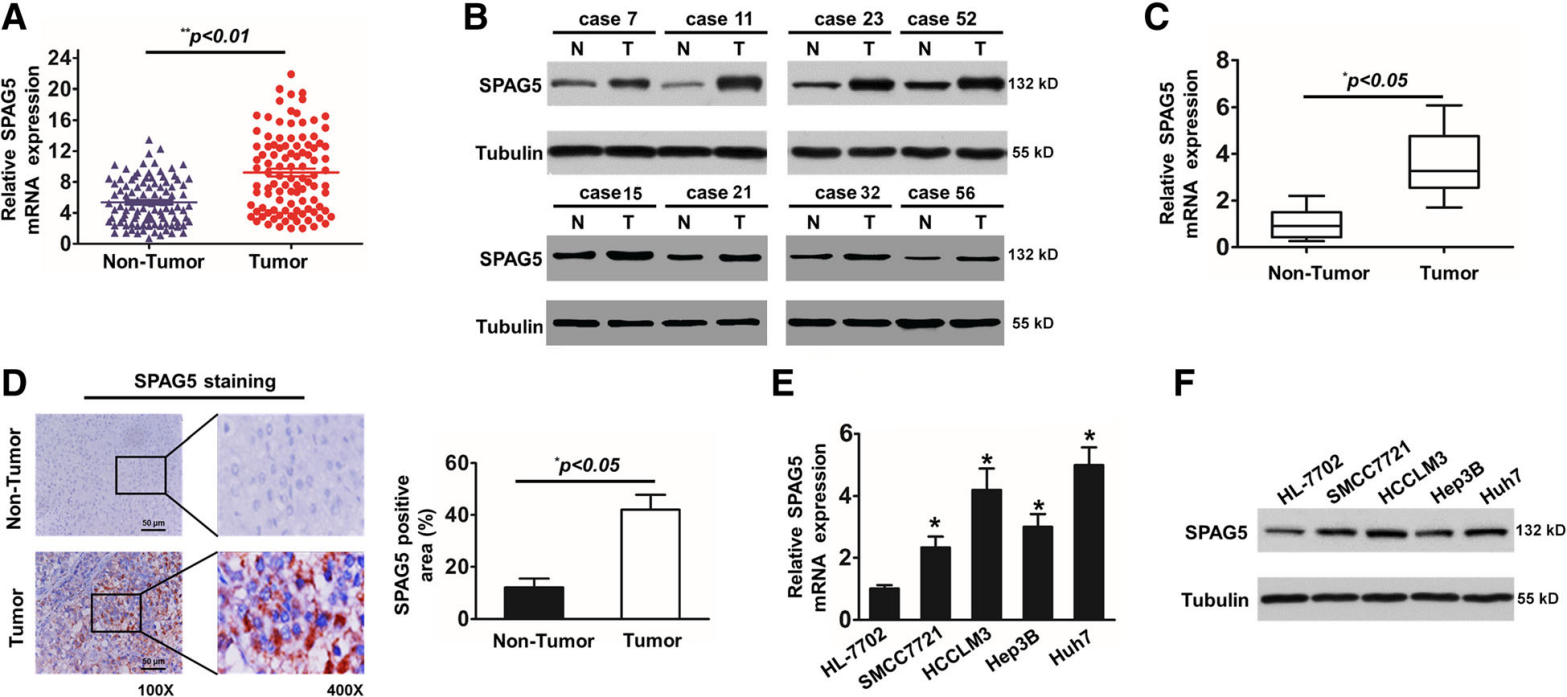
Relative SPAG5 expression in HCC tissues and HCC cells. **a**, Relative mRNA levels of SPAG5 in HCC tissues and in paired non-tumor tissues. SPAG5 expression was examined by qPCR and normalized to GAPDH expression. Statistical differences between samples were analyzed with paired samples t-test (*n* = 136, ^**^p < 0.01). **b** and **c**, Determination and quantification of SPAG5 protein levels in HCC tissues (*n* = 60) and in paired non-tumor tissues (*n* = 60) by western blotting. Tubulin was used as a loading control. **d**, Representative images (left) and quantification (right) of SPAG5 staining in 136 paired HCC tissues. Scale bar, 50 μm. **e**, qRT-PCR analysis of SPAG5 mRNA levels in HCC cell lines (Huh7, HCCLM3, SMCC7721 and Hep3B) and normal cells (HL-7702) ^*^p < 0.05. **f**, Quantification of SPAG5 protein expression using western blot analyses in HCC cell lines and HL-7702 cell

To identify the potential roles of SPAG5 in the development and progression of hepatocellular carcinoma, we first examined SPAG5 expression in 136 hepatocellular carcinoma (HCC) tissue samples and corresponding adjacent tissues by qRT-PCR. The qRT-PCR data revealed that the average fold change of SPAG5 mRNA expression in tumor tissues was significantly higher than that in paired nontumor tissues (Fig. 1a) (*p* < 0.01). Furthermore, the protein levels from western blot analysis were quantified by densitometry among 136 HCC tumors and their matched non-tumor and para-tumor tissues. Our results also showed that the SPAG5 protein levels were significantly elevated in HCC tissues compared to adjacent normal tissue (Fig. 1b-c). In addition, the levels of SPAG5 protein in the cancer tissue samples and corresponding adjacent tissues were investigated by IHC with an anti-SPAG5 antibody. The IHC results showed that the SPAG5 protein was highly expressed in 68.4% (93 of 136) of the HCC tissue samples, which was consistent with the western blot results (Fig. 1d). These results indicated that the expression of SPAG5 was significantly upregulated in HCC tissues.

Next, to understand the expression of SPAG5 in HCC cells, we investigated the expression of SPAG5 by qRT-PCR in four human HCC cell lines. Our results showed that the expression levels of SPAG5 in HCC cells were higher than the levels in normal liver cells (HL7702). Huh7 showed the highest expression level of SPAG5, which was ~ 5 times that of HL7702. HCCLM3, SMCC7721, and Hep3B cells showed ~ 4.2, ~ 2.2, and ~ 2.8 times higher expression levels of SPAG5 than HL7702, respectively (Fig.1e). To confirm the protein expression of SPAG5 in these cell lines, western blot analysis was performed, and our results also showed that SPAG5 expression in HCC cells was higher than in normal liver cells (Fig. 1f). Taken together, these data suggest that the expression of SPAG5 were significantly upregulated in HCC tissues and cells.

### High SPAG5 expression affects prognosis of HCC patients

**Fig. 2:**
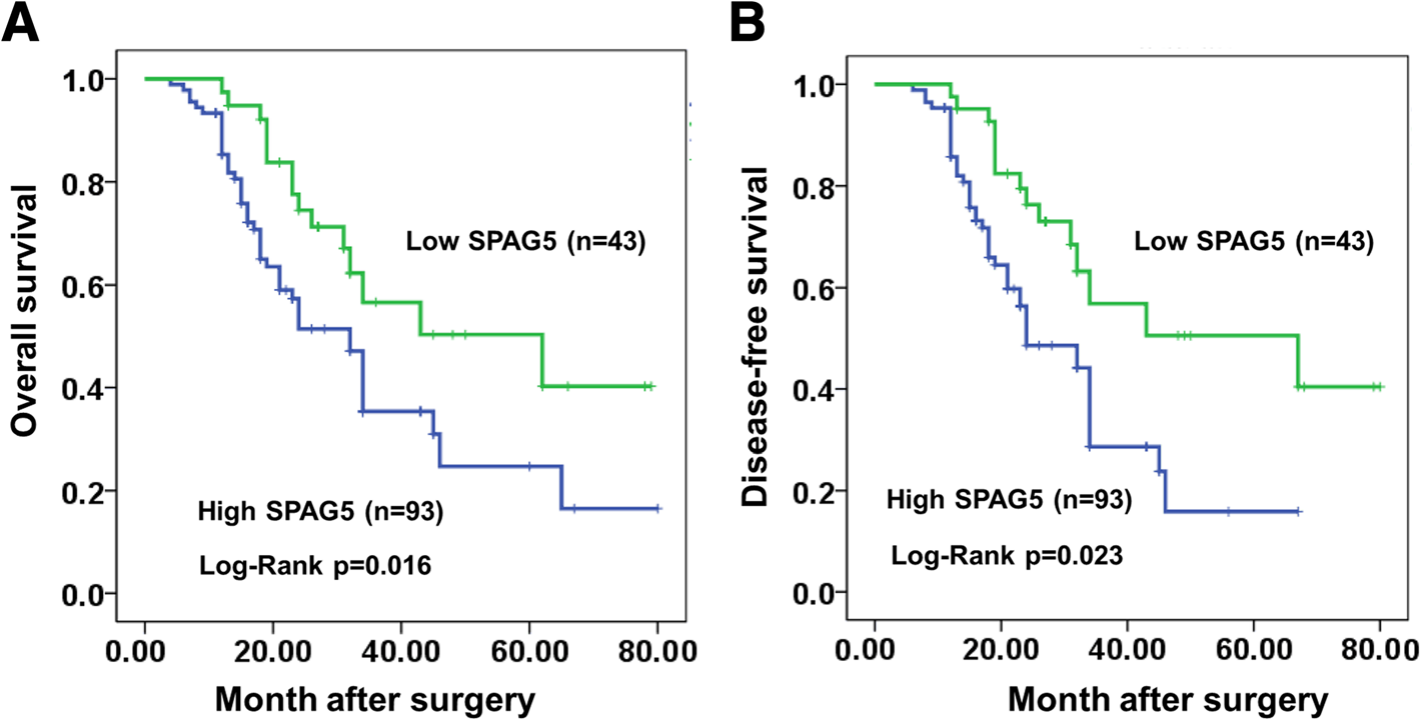
Relative SPAG5 expression and its clinical significance. **a** and **b**, Kaplan-Meier curves for overall survival (OS) and disease-free survival (DFS) of two groups defined by low (*n* = 43) and high (*n* = 93) expression of SPAG5 in patients with HCC. ^*^p < 0.05

To investigate the correlation between SPAG5 overexpression and HCC clinicopathological parameters, the expression of SPAG5 was examined by IHC in sections from HCC specimens along with the clinicopathological features. The analysis of the clinical features of HCC patients revealed that SPAG5 overexpression was closely correlated with Tumor size (*p* < 0.01), but was not significantly correlated with Age, Gender, Tumor Number, and Tumor location (Table 1). Furthermore, we found that the SPAG5 protein levels were associated with the prognosis of HCC patients. Kaplan-Meier analysis revealed that high SPAG5 protein expression was significantly associated with poor overall survival (OS) in HCC patients (*p* < 0.05) (Fig. 2a). In addition, disease-free survival (DFS) in the high SPAG5 expression group than those in the low expression group were observed (Fig. 2b, *p* < 0.05). Moreover, Univariate analysis showed that tumor size and high SPAG5 protein expression (*p* < 0.01) were significantly associated with poor OS. Multivariate analyses further revealed that high SPAG5 expression was one of the independent predictive factors for poor OS in HCC (Table 2). Overall, these results indicate that SPAG5 overexpression is positively correlated with the HCC progression and is indicative of poor prognoses.

**Table 1:** Relationship between SPAG5 expression and clinicopathological features in 136 HCC patients

Parameters	Total case	SPAG5		*P* value
	136	High expression (N=93)	Low expression (N=43)	
Age(years)				P=0.845
<60	87	60	27	
≥60	49	33	16	
Gender				P=0.939
Female	29	20	9	
Male	107	73	34	
Tumor size (cm)				***P<0.001***
<5	70	33	37	
≥5	66	60	6	
Tumor nodule number				P=0.499
Single	100	70	30	
Multiple	36	23	13	
HBsAg				P=0.939
Negative	29	20	9	
Positive	107	73	34	
AFP(ng/ml)				***P<0.001***
<400	39	6	33	
≧400	97	87	10	
Cirrhosis				P=0.601
Absence	108	75	33	
Presence	28	18	10	
Liver function				P=0.804
Child-Pugh A	109	74	35	
Child-pugh B	27	19	8	
Lobe				P=0.222
Right	95	68	27	
Left	41	25	16	
TNM				P=0.813
I/II	93	63	30	
III/IV	43	30	13

**Table 2:** Univariate and multivariate analyses of overall survival in HCC patients

Parameters	Univariate analysis			Multivariate analysis		
	HR	95%CI	*P* value	HR	95%CI	*P* value
Age (≥60/ <60)	0.799	0.621-1.332	0.827	—	—	—
Gender(Male/Female)	1.032	0.761-1.753	0.239		—	—
Tumor nodule number (Single/Multiple)	0.916	0.755-1.612	0.713	—	—	—
HBsAg (Negative/Positive)	0.713	0.473-1.241	0.542	—	—	—
TNM( I,II /III,IV)	1.130	0.543-1.564	0.712	—	—	—
Cirrhosis (Absence/Presence)	1.342	0.812-1.945	0.423	—	—	—
Liver function (Child-Pugh A/Child-pugh B)	0.974	0.728-1.863	0.513	—	—	—
Lobe (Right/Left)	0.863	0.673-1.752	0.631	—	—	—
AFP(ng/ml) ( <400/≧400)	1.193	1.108-2.109	0.021^*^	2.121	1.532-2.896	0.034^*^
Tumor size (<5/≥5)	1.729	1.215-2.651	0.014^*^	2.154	1.959-3.712	0.042^*^
SPAG5 expression (High/Low)	1.828	1.429-2.744	0.017^*^	1.970	1.786-3.021	0.021^*^

^*^P<0.05

### Knockdown of SPAG5 inhibits HCC tumor growth in vivo and in vitro

**Fig. 3:**
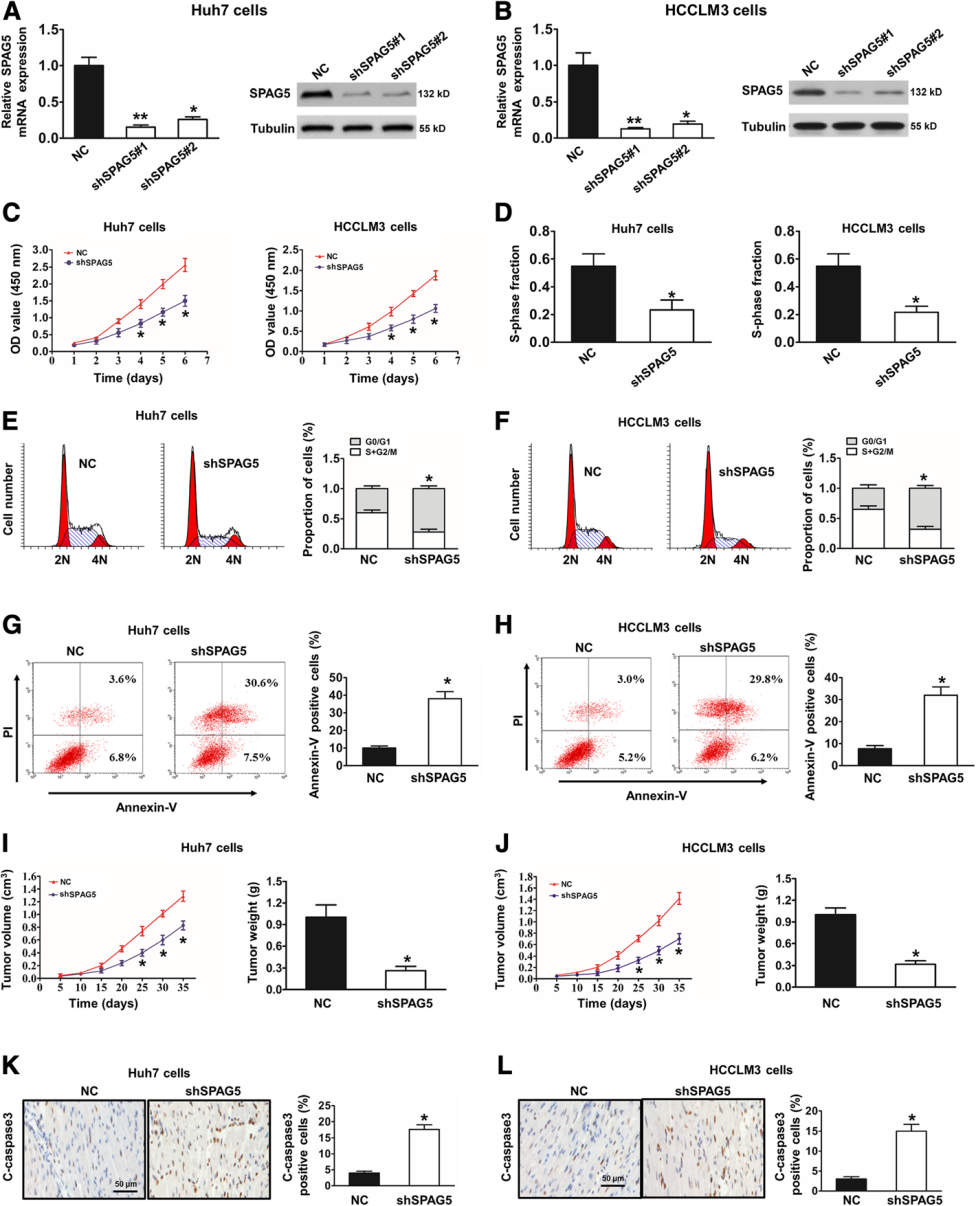
SPAG5 promotes HCC proliferation in vitro and in vivo. **a** and **b**, The efficiency of SPAG5 silencing in HCC cell lines was measured by RT-PCR and Western blot. **c**, The proliferation capacities were detected by CCK8 assays in Huh7 and HCCLM3 cells transfected with the shNC or the shSPAG5 plasmid ^*^p < 0.05. **d**, EdU results showing the effect of SPAG5 knockdown on the proliferation abilities of Huh7 and HCCLM3 cells, ^*^p < 0.05. **e** and **f**, Detection for cell cycle of HCC cells after silencing SPAG5 expression in Huh7 and HCCLM3 cells. Results are expressed as peak diagram (left) and calculated distribution for cells in G0/G1, S, and G2/M phases (right). ^*^p < 0.05. **g** and **h**, Measurement of apoptotic cells under SPAG5 down-regulation in Huh7 (**g**) and HCCLM3 cells (**h**). Results are expressed as scatter diagram (left) and calculated percentage of annexin-V-positive cell population (right). ^*^p < 0.05. **i** and **j**, The in vivo tumor formation was examined by subcutaneously injecting shNC or shSPAG5 cells into the flank of nude mice (n = 6), ^*^p < 0.05. **k** and **l**, representative examples of the immunohistochemistry analyses of xenograft tumors with anti-C-caspase-3 antibodies. Scale bar, 50 μm. Quantifcation of anti-C-caspase-3–positive cells of three individual tumors. ^*^p < 0.05

To investigate the potential biological function of SPAG5 in HCC development, we stably transfected a SPAG5-specific short hairpin RNA (shSPAG5) into Huh7 and HCCLM3cells, and the western blot results showed that decreasing SPAG5 transcript levels significantly reduced SPAG5 protein expression in Huh7 and HCCLM3 cells (Fig. 3a-b). In addition, to rule out possible off-target effects associated with shRNAs, we generated a SPAG5 cDNA harboring silent mutations in the shRNA-targeting sequence that made the mRNA insensitive to this shRNA (Additional file 1: Figure S1A and B). As shown in Fig. 3c, the CCK8 assay further verified that downregulation of SPAG5 inhibits cell proliferation in Huh7 and HCCLM3 cell lines compared with the NC group. Furthermore, using a 5-ethynyl-20-deoxyuridine (EdU) assay, we found that downregulation of SPAG5 inhibits the growth rate of HCC cells compared with the NC group (Fig. 3d). Moreover, Flow cytometry showed that downregulation of SPAG5 significantly arrested the cell cycle at the G1 phase in Huh7 and HCCLM3 cells (Fig. 3e and f). In additon, as shown in Fig. 3g and h, our results showed that the percentage of apoptotic cells was significantly increased in the SPAG5 knockdown group compared with the control group. These data collectively indicate that knockdown of SPAG5 exerted tumor-suppressive effects in human HCC cells in vitro. To further investigate SPAG5 influence on HCC tumor growth in vivo, we performed the tumorigenicity assay in nude mice. The results indicated that the subcutaneous tumors of the shSPAG5 group had a smaller volume and lower weight than those of the NC group, demonstrating that the reduction in SPAG5 significantly inhibited tumor growth (*p* < 0.05) (Fig. 3i and j). Furthermore, knockdown of SPAG5 tumor cells exhibited enhanced apoptosis (Fig. 3k and l). Taken together, these data indicated that knockdown of SPAG5 inhibits HCC tumor growth in vivo and in vitro*.*

### Expression levels of SPAG5 and SCARA5 are inversely correlated in HCC cells and tissues

**Fig. 4:**
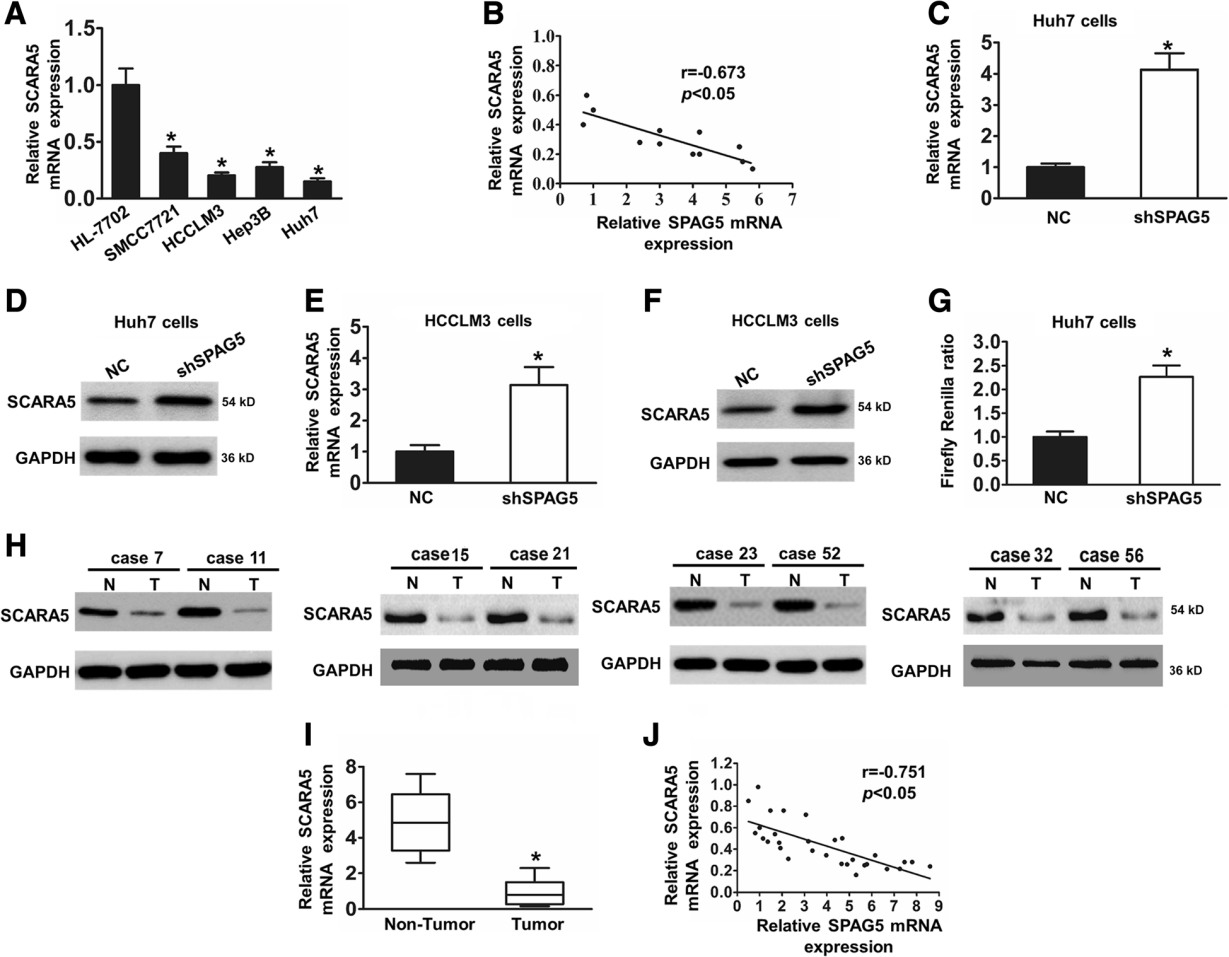
Stable knockdown of SPAG5 reduced SCARA5 expression in HCC cells. **a**, qRT-PCR analysis of the expression of SCARA5 in HCC cells and HL-7702 cells. ^*^p < 0.05. **b**, Scatter plots showing the negative linear correlation between the mRNA expression of SPAG5 and that of SCARA5 in HCC cells. **c**-**f**, qRT-PCR and Western blot analyses was performed to detect the SCARA5 expression levels in Huh7 cells (**c** and **d**) and HCCLM3 cells (**e** and **f**) stably transfected with the shNC vector or the shSPAG5 plasmid. ^*^p < 0.05. **g**. Luciferase analysis for SCARA5 promoter activity in SPAG5-knockdown HCC cells. ^*^p < 0.05. **h**, Determination of SPAG5 protein levels in HCC tissues (*n* = 60) and in paired non-tumor tissues (*n* = 60) by western blotting. Tubulin was used as a loading control. **i**, Relative mRNA levels of SCARA5 in HCC tissues (*n* = 136) and in paired non-tumor tissues (*n* = 136). **j**, scatter plots show a positive correlation between SPAG5 and SCARA5 at the mRNA level in HCC

Then, we wanted to further investigate the mechanism by which SPAG5 regulates HCC tumor growth. Recently, our previous and other studies have revealed that SCARA5 knockdown enhances tumor growth in HCC [17, 19]. Therefore, we speculated that SPAG5 might influence HCC progression by regulating SCARA5 expression. To investigate the relationship between SPAG5 and SCARA5, we first detected the expression levels of SCARA5 in various HCC cells by qRT-PCR. The data of qRT-PCR showed that the mRNA level of SPAG5 was negatively correlated with the mRNA level of SCARA5 in HCC cells (Fig. 4a-b). Next, we manipulated the expression levels of SPAG5 by stably transfecting cells with shSPAG5, and we examined the expression of SPAG5 and SCARA5 by qRT-PCR and western blotting analysis in Huh7 and HCCLM3 cells. The western blotting and qRT-PCR analyses showed that the downregulation of SPAG5 significantly increased SCARA5 expression levels in Huh7 and HCCLM3 cells (Fig. 4c-f). Our results also demonstrate that co-transfection of pmirGLO-SCARA5 and shSPAG5 (pmirGLO-SCARA5 + shSPAG5 group) obviously decreased the luciferase activity compared with that in pmirGLO-SCARA5 + shNC group (Fig. 4g). In addition, we determined the expression levels of SPAG5 and SCARA5 in HCC tissue and adjacent tissues. The results revealed the SCARA5 protein and mRNA expression was significantly decreased in HCC tissues (Fig. 4h and i).Moreover, the scatter plots showed that the SPAG5 and SCARA5 mRNA expression levels were inversely correlated in HCC tissues (Fig. 4j). Taken together, these results indicate that SPAG5 influences HCC progression by regulating SCARA5 expression.

### SCARA5 is crucial for SPAG5-mediated HCC progression in vitro and in vivo

**Fig. 5:**
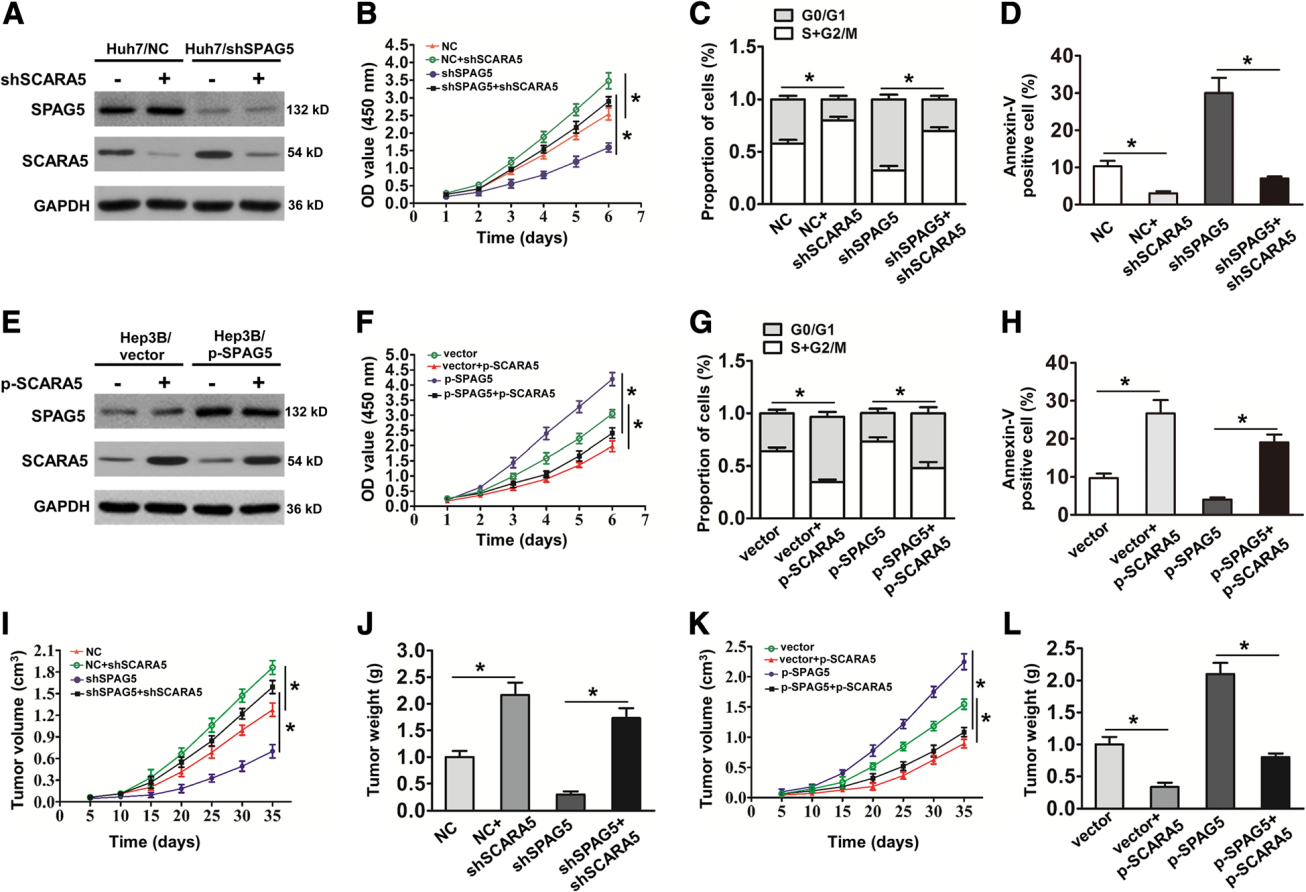
SCARA5 is key for SPAG5-mediated HCC proliferation. **a**, The knockdown of SCARA5 expression markedly inhibited the increase in SCARA5 expression observed in Huh7/shSPAG5 cells. **b** and **c**, CCK-8 assay and Flow Cytometry assay showed that SCARA5 inhibition rescued the decreased cell proliferation ability of Huh7/shSPAG5 cells. ^*^p < 0.05. **d**, results are expressed as calculated percentage of Annexin-V-positive cell population. ^*^p < 0.05. **e**. Western blotting was used to detect the expression of SPAG5 and SCARA5. The overexpression of ectopic SCARA5 attenuated the loss of SCARA5 expression in Hep3B/p-SPAG5 cells. **f** and **g**, CCK-8 assay and Flow Cytometry assay showed that the overexpression of ectopic SCARA5 significantly reduced the SPAG5-enhanced cell proliferation observed in Hep3B/p-SPAG5 cells. ^*^p < 0.05. **h**, results are expressed as calculated percentage of Annexin-V-positive cell population. ^*^p < 0.05. **i**–**l**, The tumor sizes and tumor weight were measured in each groups. ^*^p < 0.05

To further validate that SPAG5 mediates HCC progression by regulating SCARA5, we first inhibited the expression of SCARA5 in SPAG5-knockdown HCC cells and then observed SPAG5 and SCARA5 protein expression levels and cell proliferation. As shown in Fig. 5a, the western blotting results showed that the downregulation of SCARA5 markedly inhibited the increase of SCARA5 expression in SPAG5-knockdown Huh7 cells. Simultaneously, the downregulation of SCARA5 rescued the decreased proliferation and increased apoptosis induced by SPAG5 knockdown (Fig. 5b-d). In contrast, overexpression of SCARA5 markedly attenuated the loss of SCARA5 expression, significantly reduced cell proliferation and decreased apoptosis in SPAG5-overexpressing Hep3B cells (Fig. 5e-h). Furthermore, a tumorigenicity assay also showed that downregulation of SCARA5 increased the volumes and weights of the Huh7/shSPAG5 group (Fig. 5i and j), whereas upregulation of SCARA5 decreased the volumes and weights of the Hep3B/SPAG5 group (Fig. 5k and l). Thus, these results demonstrated that SCARA5 is crucial for SPAG5-mediated HCC cell progression in vitro and in vivo*.*

### SPAG5 regulates SCARA5 expression through modifying β-catenin degradation

**Fig. 6:**
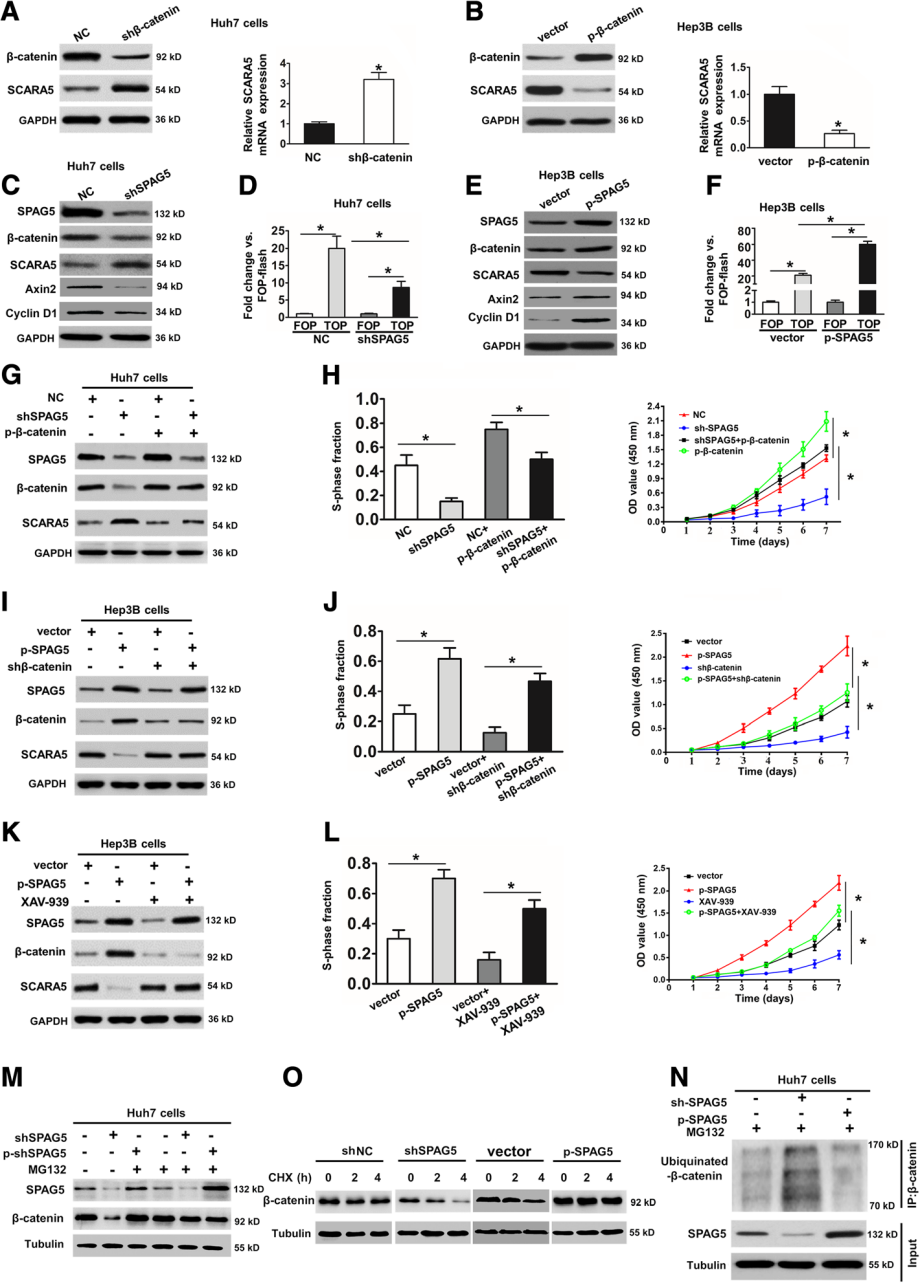
SPAG5 regulates SCARA5 expression through the wnt/β-catenin pathway in HCC cells. **a**, Western blot and qRT-PCR analyses were used to detect SCARA5 expression in Huh7 cells stably transfected with the shNC or the shβ-catenin plasmid. ^*^p < 0.05. **b**, Western blot analyses were used to detect SCARA5 expression in Hep3B cells stably transfected with the vector or the p-β-catenin plasmid. ^*^p < 0.05. **c**, Huh7 cells transfected with shNC and shSPAG5 were subjected to western blot analyses for the indicated proteins. **d**, the relative luciferase activity levels in cells transfected with TOP-flash and FOP-flash vectors in Huh7 cells transfected with shNC and shSPAG5 are shown. ^*^p < 0.05. **e**, Hep3B cells transfected with vector and p-SPAG5 were subjected to western blot analyses for the indicated proteins. **f**, the relative luciferase activity levels in cells transfected with TOP-flash and FOP-flash vectors in Hep3B cells transfected with vector and p-SPAG5 are shown. ^*^p < 0.05. **g** and **h**, Huh7 cells were transfected with the indicated plasmid. The quantity of SPAG5, β-catenin and SCARA5 were assessed by western blot analysis. The cancer cells’ proliferation capacities were detected by EdU and CCK8 assays. ^*^p < 0.05. **i** and **j**, Hep3B cells were transfected with the indicated plasmid. The quantities of SPAG5, β-catenin and SCARA5 were assessed by western blot analysis. The proliferation capacities of the cancer cells were detected by EdU and CCK8 assays. ^*^p < 0.05. **k** and **l**, Hep3B cells transduced with vector and p-SPAG5 plasmid were treated with XAV-939. The quantities of SPAG5, β-catenin and SCARA5 were assessed by western blot analysis. The proliferation capacities of the cancer cells were detected by EdU and CCK8 assays. ^*^p < 0.05. **m**. cells transduced with SPAG5 shRNA or p-SPAG5 plasmid were treated with 10 μM MG132. Cells were collected at 6 h and immunoblotted with the antibodies indicated. **n**. Huh7 cells were transfected with SPAG5 shRNA or p-SPAG5 plasmid, and treated with cycloheximide (CHX). Cells were collected at different time points and immunoblotted with the antibodies indicated. **o**. Lysates from Huh7 cells transduced with SPAG5 shRNA or p-SPAG5 plasmid were immunoprecipitated with the anti-Ub and immunoblotted with the anti-β-catenin. Cells were treated with MG132 for 6 h before collection

Many studies have demonstrated that the β-catenin/TCF4 pathway plays a critical role in regulating cancer cell proliferation [20, 21]. In addition, β-catenin could regulate SCARA5 expression in RCC cell [18]. Therefore, we investigated whether β-catenin/TCF4 signaling is involved in SPAG5 regulation of SCARA5 in HCC. To test this hypothesis, we first measured the changes in SCARA5 expression in the β-catenin knockdown Huh7 cells. The results showed that the knockdown of β-catenin can significantly increase SCARA5 mRNA and protein expression in Huh7 cells (Fig. 6a), whereas β-catenin downregulation again had the opposite effect in HCC cells (Fig. 6b). These findings demonstrated that β-catenin regulates SCARA5 expression in HCC cells. Next, we aimed to elucidate whether SPAG5 affects expression of SCARA5 via β-catenin, we further measured the changes in β-catenin and SCARA5 expression in the SPAG5 knockdown Huh7 cells. The results showed that the knockdown of SPAG5 can significantly decrease the β-catenin and increased SCARA5 protein expression in Huh7 cells (Fig. 6c). Besides, we also examined the β-catenin transcriptional activity in HCC cells after SPAG5 silencing. In a TOP-Flash reporter luciferase assay, the knockdown of SPAG5 in Huh7 cells decreased the transcriptional activity of TCF4 compared with the control groups (Fig. 6d). In contrast, overexpression of SPAG5 can significantly increase the β-catenin and decreased SCARA5 protein expression in SMCC7721 cells (Fig. 6e). Additionally, we found that SPAG5 silencing significantly inhibited the expression levels of Axin2 and Cyclin D1 in Huh7 cells (Fig. 6c), whereas overexpression of SPAG5 can increased the Axin2 and Cyclin D1 protein expression in SMCC7721 cells (Fig. 6e). Furthermore, overexpression of SPAG5 in Hep3B cells increased the transcriptional activity of TCF4 compared with the control groups (Fig. 6f). These studies showed that the wnt/β-catenin pathway is involved in SPAG5 regulation of SCARA5 expression.

**Fig. 7:**
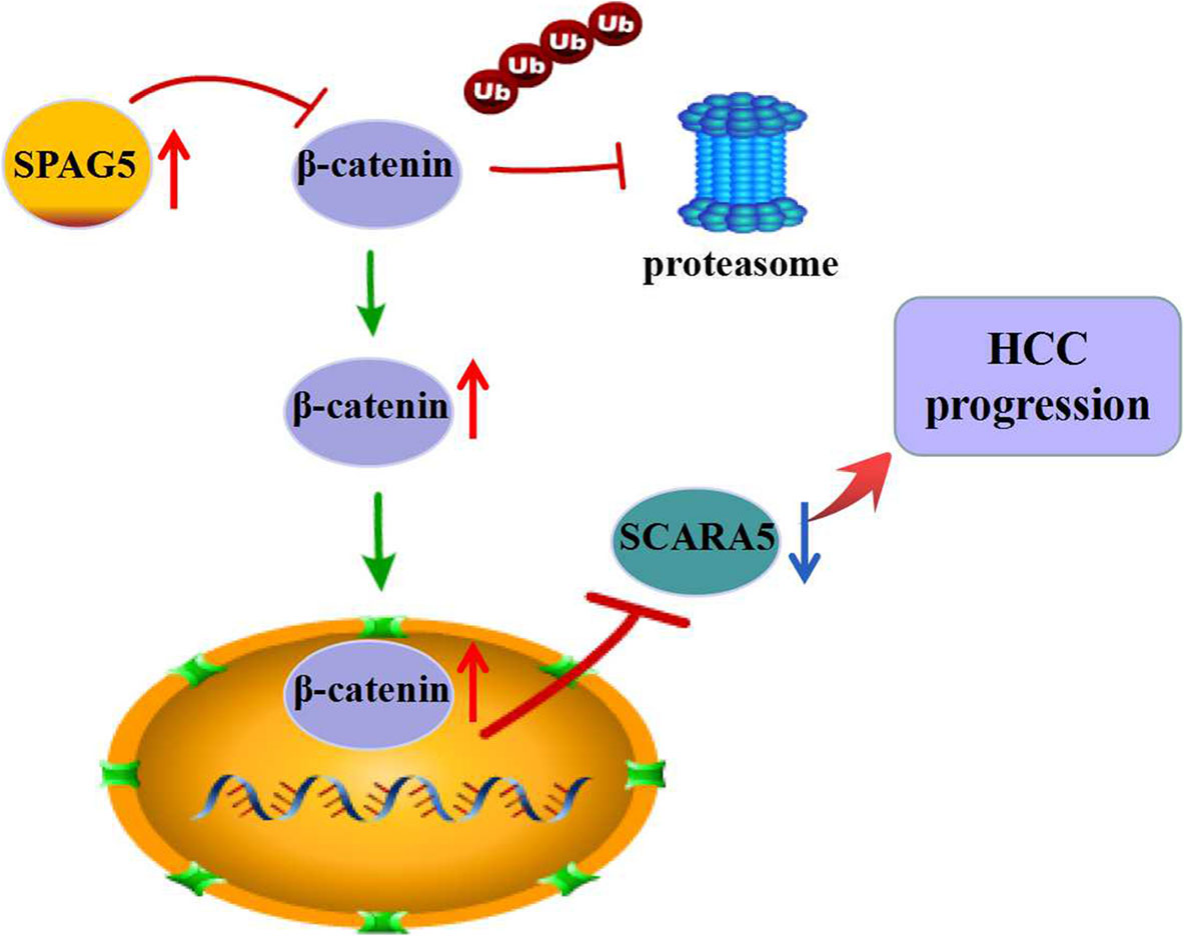
Proposed model by which SAPG5 promotes HCC progression by SCARA5 inhibition through modifying β-catenin ubiquitination

To further verify that SPAG5 regulates SCARA5 expression through the β-catenin pathway in HCC cells, we first overexpressed β-catenin in SPAG5-downregulated HCC cells. The results showed that the overexpression of ectopic β-catenin inhibited the increases in SCARA5 expression and the cell proliferation ability was also rescued in SPAG5-knockdown Huh7 cells (Fig. 6g-h).We then knocked down β-catenin in SPAG5-overexpressing HCC cells and found that the downregulation of β-catenin could rescue the decreased SCARA5 expression in Hep3B/SPAG5 cells. The HCC cell proliferation ability was also decreased (Fig. 6i-j). In addition, we increased SPAG5 expression in HCC cells by treating them with specific inhibitor of β-catenin, XAV-939 (Tankyrase 1/2 inhibitor), which selectively inhibits β-catenin-mediated transcription through promoting β-catenin degradation. The results showed that overexpression of SPAG5 had no effect on SCARA5 protein and mRNA expression when β-catenin was inhibited (Fig. 6k-l). The above mentioned results demonstrated that SPAG5 regulates SCARA5-induced HCC proliferation depend on the wnt/β-catenin pathway.

Finally, we further assessed the mechanisms through which SPAG5 regulates β-catenin. Our results showed that the expression of β-catenin protein was no difference after dysregulation of SPAG5 in the presence of the proteasome inhibitor MG132 (Fig. 6m). These findings confirmed that SPAG5 regulates β-catenin protein levels through ubiquitin–proteasome system (UPS)-mediated manner in HCC cells. To further investigate whether SPAG5 regulates the stability of β-catenin protein, we evaluated β-catenin protein levels in the presence of cycloheximide (CHX), an inhibitor of translation. Notably, overexpression of SPAG5 in Huh7 cells led to a pronounced increase in β-catenin protein stability. Conversely, SAPG5 silencing in Huh7 cells reduced β-catenin stability (Fig. 6n). Meanwhile, our data indicate that knockdown of CSN5 dramatically increased the levels of β-catenin ubiquitination, while overexpression of SAPG5 significantly decrease the levels of β-catenin ubiquitination (Fig. 6o). Therefore, these results confirmed that SAPG5 promotes HCC progression by SCARA5 inhibition through modifying β-catenin ubiquitination.

## Discussion

Hepatocellular carcinoma (HCC) is the third leading cause of cancer-related deaths globally [22, 23]. Many studies have demonstrated that HCC progression is closely related to abnormal expression of oncogenes genes. Recently, some studies reported that SPAG5 was amplified at the 17q11 region in some types of cancer and involved in various fundamental biological processes including cell proliferation [24, 25]. There has been accumulating evidence that shows that abnormally high expression of SPAG5 is closely related to cancer, and SPAG5 could serve as a novel cancer biomarker [10]. However, no information is currently available on the specific role or molecular mechanism of SPAG5 in HCC patients. In the present study, our findings showed that SPAG5 expression remarkable increase in HCC tissues compared to the corresponding non-tumor tissues. Furthermore, we revealed that high expression level of SPAG5 is closely associated with cancer phenotypes, including tumor size. Multivariate analyses further revealed that high SPAG5 expression was an independent predictive factor for poor OS in HCC. Moreover, function assays revealed that SPAG5 downregulation led to a marked inhibition of proliferation in vitro, and suppression of tumor growth in vivo. In addition, knockdown of SPAG5 tumor cells exhibited enhanced apoptosis in vitro and in vivo*.* Taken together, these data indicate that SPAG5 may function as an oncogene and might play an important role in HCC development and progression.

Next, we explored the mechanism by which SPAG5 regulates HCC progression. Recently, the role of SCARA5 in tumor development has attracted much attention. SCARA5 is a scavenger receptor, and SCARA5 levels are significantly lower in glioma and non-small cell lung cancer tissues compared with normal tissue [14–16]. The upregulation of SCARA5 expression significantly suppresses cell proliferation in glioma cells. Thus, SCARA5 was identified as a candidate tumor suppressor gene. Our previous studies have also demonstrated that SCARA5 knockdown enhances cancer cell progression in HCC [17]. Herein, we reveal a novel mechanism that underlies the inhibition of HCC progression, which occurs through an increase in SCARA5 expression mediated by SPAG5 silencing. First, we found that the SPAG5 expression levels are high in HCC tissues and that the SCARA5 expression levels are low in HCC tissues. The expression levels of SPAG5 and SCARA5 were found to be negatively correlated. Furthermore, our data demonstrated that the downregulation of SPAG5 expression increased SCARA5 expression and inhibited HCC progression. Moreover, SCARA5 downregulation rescued the decreased cell progression induced by SPAG5 knockdown, whereas SCARA5 upregulation significantly decreased SPAG5-enhanced cell progression. Overall, these results demonstrated that SPAG5 regulates SCARA5 expression to influence HCC progression, identifying a new regulatory mechanism of SCARA5.

Finally, we further investigated the molecular mechanism by which SPAG5 regulates SCARA5 expression. Research has demonstrated that the β-catenin/TCF4 pathway plays a critical role in regulating HCC progression, where β-catenin is the key transducer of Wnt signaling [26–28]. Importantly, research has demonstrated that β-catenin/TCF4-SCARA5 axis plays an important role in the progression of renal cell carcinoma (RCC) [18]. Here, we reveal a novel mechanism by which SPAG5 regulates SCARA5 expression by activating the Wnt/β-catenin signaling pathway. This conclusion is based on the following observations. First, our results showed that the knockdown of β-catenin can significantly increase SCARA5 mRNA and protein expression in HCC cells. Second, overexpression of SPAG5 can significantly increase the β-catenin and decreased SCARA5 protein expression, and increased the transcriptional activity of TCF4 compared with the control groups. Third, the knockdown of SPAG5 increased SCARA5 expression, whereas upregulation of β-catenin could rescue the increased SCARA5 expression levels induced by SPAG5 knockdown. Furthermore, overexpression of SPAG5 had no effect on SCARA5 expression after the addition of specific inhibitors of β-catenin. Taken together, these data demonstrate that SPAG5 regulates SCARA5-induced HCC progression via β-catenin/TCF4 pathway.

Studies have shown that posttranslational modifications are involved in regulating β-catenin expression, including ubiquitination [29, 30]. For instance, knockdown of EGF could inhibit prostate cancer cell EMT by promoting β-catenin ubiquitination. Importantly, in this study, our results have suggested for the first time that the SPAG5 pathway negatively regulates β-catenin protein ubiquitination and degradation. This conclusion is based on the following observations. First, SPAG5 can increase the half-life of β-catenin. Second, overexpression of SPAG5 in HCC cells led to a pronounced increase in β-catenin protein stability. Conversely, SAPG5 silencing in HCC cells reduced β-catenin stability. Third, the knockdown of SPAG5 could promote levels of β-catenin ubiquitination, whereas increased SPAG5 significantly reduced the levels of β-catenin ubiquitination. Additionally, β-catenin is a transcriptional coactivator that promotes target gene expression. Recent studies have demonstrated that knockdown of β-catenin reduced TCF4 expression and increased SCARA5 expression, whereas the overexpression of β-catenin increased TCF4 expression and downregulated SCARA5 expression [17, 18]. In line with the previous study, our findings also revealed that knockdown of SPAG5 obviously upregulated the expression of SCARA5 through reducing the expression level of β-catenin. However, the detailed molecular mechanism needs further study in the future.

## Conclusions

In conclusion, our results highlight the crucial role played by SPAG5 in HCC proliferation and find the overexpression of SPAG5 can down-regulates SCARA5 expression through β-catenin/TCF4 signaling pathway in HCC cells (Fig.7). Thus, SPAG5 could serve as a candidate biomarker for the future diagnosis and therapy of HCC.

## Additional file

Additional file 1: **Figure S1.** shRNA-resistant SPAG5 plasmid resuces the expression of SPAG5. (TIF 6686 kb)

## Abbreviations

CCK-8: Cell counting kit-8
DFS: Disease-free survival
Edu: 5-Ethynyl-20-deoxyuridine
HCC: Hepatocellular carcinoma
OS: Poor overall survival
SCARA5: Scavenger receptor class A member 5
SPAG5: Sperm-associated antigen 5
