# Supplementary figures and images for: SPAG5 promotes hepatocellular carcinoma progression by downregulating SCARA5 through modifying β-catenin degradation
Source: J Exp Clin Cancer Res. 2018 Sep 18;37:229. doi: 10.1186/s13046-018-0891-3 (PMC6154423; doi:10.1186/s13046-018-0891-3)

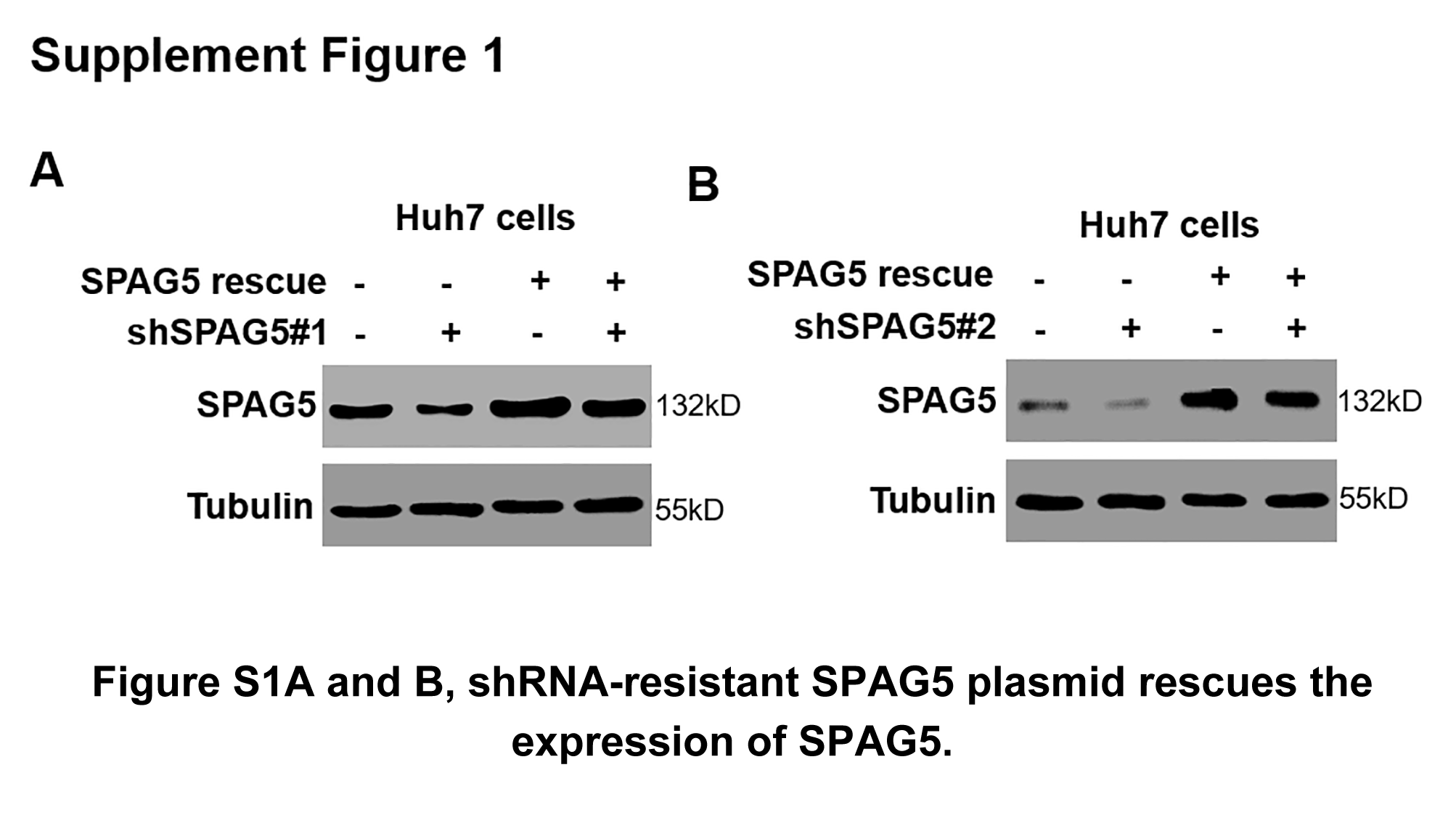

Supplement: Supplementary file 1 — Figure S1. shRNA-resistant SPAG5 plasmid resuces the expression of SPAG5. (TIF 6686 kb) [file 13046_2018_891_MOESM1_ESM.tif]
